# [Retracted] Targeting of TLE3 by miR‑3677 in human breast cancer promotes cell proliferation, migration and invasion

**DOI:** 10.3892/ol.2024.14840

**Published:** 2024-12-09

**Authors:** Li-Na Peng, Xing-Yan Deng, Xiao-Xiong Gan, Jin-Hui Zhang, Guang-Hui Ren, Fei Shen, Jian-Hua Feng, Wen-Song Cai, Bo Xu

Oncol Lett 19: 1409–1417, 2020; DOI: 10.3892/ol.2019.11241

Following the publication of this paper, it was drawn to the Editor's attention by a concerned reader that certain of the Transwell migration assay data shown in Figs. 2D and 3D had already appeared in a number of previously published articles written by different authors at largely different research institutes. Owing to the fact that the contentious data in the above article had already been published prior to its submission to *Oncology Letters*, the Editor has decided that this paper should be retracted from the Journal. The authors were asked for an explanation to account for these concerns, but the Editorial Office did not receive a satisfactory reply. The Editor apologizes to the readership for any inconvenience caused.

